# COVID-19-Related Anosmia: The Olfactory Pathway Hypothesis and Early Intervention

**DOI:** 10.3389/fneur.2020.00956

**Published:** 2020-09-10

**Authors:** Alessandra Gori, Fabrizio Leone, Lorenzo Loffredo, Bianca Laura Cinicola, Giulia Brindisi, Giovanna De Castro, Alberto Spalice, Marzia Duse, Anna Maria Zicari

**Affiliations:** ^1^Department of Pediatrics, Sapienza University, Rome, Italy; ^2^Department of Internal Medicine and Medical Specialties, Sapienza University, Rome, Italy; ^3^Child Neurology Division, Department of Pediatrics, Sapienza University of Rome, Rome, Italy

**Keywords:** COVID-19, anosmia, SARS-CoV-2, olfactory receptors, olfactory bulb

## Abstract

Anosmia is a well-described symptom of Corona Virus Disease 2019 (COVID-19). Several respiratory viruses are able to cause post-viral olfactory dysfunction, suggesting a sensorineural damage. Since the olfactory bulb is considered an immunological organ contributing to prevent the invasion of viruses, it could have a role in host defense. The inflammatory products locally released in COVID-19, leading to a local damage and causing olfactory loss, simultaneously may interfere with the viral spread into the central nervous system. In this context, olfactory receptors could play a role as an alternative way of SARS-CoV-2 entry into cells locally, in the central nervous system, and systemically. Differences in olfactory bulb due to sex and age may contribute to clarify the different susceptibility to infection and understand the role of age in transmission and disease severity. Finally, evaluation of the degree of functional impairment (grading), central/peripheral anosmia (localization), and the temporal course (evolution) may be useful tools to counteract COVID-19.

## Introduction

A wide spectrum of symptoms characterizes SARS-CoV-2 infection, ranging from serious conditions, including acute respiratory distress syndrome (ARDS), to mild/moderate and also asymptomatic forms of the disease, contributing to the spread of the viral infection.

The Corona Virus Disease 2019 (COVID-19) rapid worldwide spread has led to characterization of “minor” symptoms, such as anosmia (1).

Anosmia was underestimated in the early stages of pandemic emergency, and most of the patients who needed hospitalization were not consistently investigated for this symptom that gradually emerged as a spy feature of infection.

Several respiratory viruses may cause post-viral olfactory dysfunction (PVOD), in most cases, reversible. Seldom, this dysfunction may persist, suggesting a sensorineural central damage (2). One of the COVID-19 clinical problems is the concomitant lack of prognostic indexes that may predict the need for early intervention and preventative therapies in patients with mild symptoms.

Since the olfactory bulb (OB) is considered an immunological organ (3), its involvement could provide information on host's immunological competence in the fight against the virus entrance and the virus spread into the central nervous system.

As members of Coronaviridiae family are known to cause CNS dysfunction, it appears mandatory to understand the role of SARS-CoV-2 neurotropism in the development of clinical manifestations.

## Epidemiology

The first symptomatic characterization of COVID-19 evolved over the past months adding to major symptoms (fever, coughing, fatigue, and shortness of breath) a broad spectrum of minor symptoms. Among those, increasing olfaction disturbance (OD) observations have made that anosmia was identified as an emerging symptom and subsequently as a marker of SARS-CoV-2 infection (1, 4, 5).

Anosmia had attracted the most public interest between physicians and the general population both because of media coverage in an atmosphere of increasing and constant alarm and concern and for its potential capability of early identification of infection. For instance, in our country, after journalistic and media announcement of anosmia as a symptom of COVID-19 in March 2020, this term had a peak in search volumes on Google (6).

In scientific literature, after the first reports of olfactory and taste disorders (OTDs) in COVID-19, an increasing and rapidly evolving detailed analysis of this symptom was progressively collected to evaluate the prevalence and patterns of anosmia and its significance in the context of COVID-19.

In February 2020, a retrospective study of 214 COVID-19 patients in Wuhan (7) reported that 11 patients (5.1%) presented hyposmia and 12 patients (5.6%) presented hypogeusia. This study was an early report derived from the analysis of medical records without information on the timing of chemosensory dysfunction onset and patient conditions. When Italy became the new pandemic epicenter, these disorders appeared to be more common, particularly in the early stages of the disease, in paucisymptomatic patients and mild to moderate COVID 19 patients.

Giacomelli et al. from Sacco Hospital in Milan, in March 2020, highlight the prevalence of chemosensory dysfunction in 59 patients with laboratory-confirmed SARS-CoV-2 infection through a verbal interview. Of these, 20 (33.9%) reported at least one taste or olfactory disorder and 11 (18.6%) reported both; 20.3% presented the symptoms before hospital admission, whereas 13.5% presented during the hospital stay. Females reported OTDs more frequently than males (8).

More in-depth, a following Italian multicenter study on olfactory and gustatory function impairment in COVID-19 shows more objective data. The study cohort was composed of 161 patients in home quarantine and 184 hospitalized patients in all disease stages from asymptomatic to severe disease. Chemosensory dysfunctions have been self-reported by 74.2% of COVID-19 patients; 79.3% of these patients reported combined chemosensitive disturbances, 8.6% reported isolated olfactory disorders, and 12.1% reported isolated taste disorders. At the test time, the condition was self-reported as completely regressed in 31.3% of the patients concerning the sense of smell and in 50.4% for the taste. More interestingly, functional evaluation of patients who reported only gustatory disorders or without chemosensory dysfunction highlights mild hyposmia in an additional 10.7% of patients. Furthermore, 70% of patients who reported complete resolution proved hyposmic to an objective test. Also, the study population subgroup analysis showed a high frequency of olfactory disorders throughout the observation period, ranging between 77.4% (days 1–4) and 69.2% (days 25–35). Anosmia or severe hyposmia affected 70.9% of patients in the early stages; they improved after the first 10 days, reaching moderate hyposmia values. Despite a more effective recovery of taste with back to normal range after 15 days, the olfactory score improved significantly in the first 2 weeks without returning to average values but always remaining in the range of hyposmia, even in the group of patients evaluated in the 3rd and 4th week from the clinical onset. Interestingly, chemosensitive symptoms were the first symptom of COVID-19 in 29.2% of patients and the only one in 9.5% of the cases. In this study, according to other previous studies, no correlation was found between olfactory and gustatory disorders and nasal obstruction or rhinitis symptoms. No significant correlation was found between the gustatory and olfactory scores and the patients' gender and age (9). In April 2020, a multicenter European study reported olfactory dysfunction (OD) in 85.6% of patients; 11.8% of these cases presented with OD onset before other symptoms. In contrast with the previous data, this study showed that males were significantly less affected by OD than females (*p* = 0.001). In addition, among the 18.2% of patients without nasal obstruction or rhinorrhea, 79.7% were hyposmic or anosmic (10).

Another cross-sectional survey on OTDs that showed a prevalence of 91% in the context of SARS-CoV-2 infection reported that females presented OTDs more frequently than males (52.6 vs. 25%, *P* = 0.036). Moreover, patients who did present at least one OTD were younger than those without OTDs (11). More recently, Chung et al. performed an observational cohort study using questionnaires and smell tests, sinus imaging, nasendoscopy, and nasal biopsies in selected patients. This study showed olfactory symptoms in 12 of 18 (67%) COVID-19 patients and OD was confirmed in six patients by BTT smell test. Computed tomography sinus, performed for the six patients with OD, found radiological evidence of sinusitis, and nasendoscopy did not find any olfactory cleft obstruction, nasal polyps, or active sinusitis. Interestingly, nasal biopsies, performed in three patients, showed minimal inflammatory changes represented by mild infiltrations of lymphocytes, plasma cells, and rare neutrophils in the stroma.

Immunofluorescence staining for CD68 established the presence of macrophages within the epithelium and the stroma. Follow-up evaluation by Butanol Threshold Test Assessment for the six patients with OD shows that OD can persist even after viral clearance in a subset of patients (12).

In conclusion, the systematic reviews show a wide variability of olfactory impairment prevalence according to the relief method of anosmia, subjective reports or objective testing or combination, and geographic location. The prevalence varies from 5.1% (7) to 98.0% (4). Interestingly, other recent reviews and studies highlighted ethnic differences in the frequency and prevalence of this chemosensory dysfunction, lower in Chinese COVID-19 patients than in Western cohorts of comparable size, attributing them to the variants of virus entry protein across populations (11, 12).

All these epidemiological data, even if conflicting due to the different assessment methods and geographic areas, could improve our knowledge of anosmia prevalence and patterns. It would be even better if methods of anosmia relief and follow-up were standardized. As noted above and in a recent case series of 86 COVID-19 patients (13), questionnaires and studies on clinical records could lead to an erroneous assessment of the prevalence of anosmia and recovery time and its subclinical persistence (9, 13).

## Physiopathology

Understanding the underlying mechanism of anosmia linked to the olfactory pathway's anatomy and physiology could help distinguish olfactory disorders into a conductive/peripheral or sensorineural/central. Conductive olfactory dysfunctions occur when mechanical barriers prevent proximity between odorants and receptors on the olfactory epithelium. On the contrary, sensorineural disorders occur due to deficient processing of odorant stimulus by the olfactory receptors (ORs), olfactory neurons, and olfactory pathways, up to the CNS (OB and olfactory brain areas). Common conductive disorders arise from obstructive nasal diseases, such as chronic rhinosinusitis, nasal polyposis, allergic rhinitis, and nasal masses, characterized by a combination of obstruction to nasal airflow transporting odorant and mucosal edema that is inflammation related (13). On the contrary, viral infections are considered the most common cause of sensorineural olfactory dysfunction. Indeed, from 20–30 to 42.5% of adult patients with acquired sensorineural olfactory dysfunction reported a recent history of upper respiratory infection (13, 14), while the cumulative frequency of olfactory loss associated with sinonasal disorders or acute infections of the upper airways was 6% in the pediatric population (15). A recent study showed that olfactory dysfunction's etiologies changed with age: the frequency of congenital causes of anosmia decreased while upper respiratory tract infection-related anosmia frequency and idiopathic causes increased (15).

It was estimated that children suffer from 6 to 10 colds per year, whereas adults from 2 to 4 per year. More than 200 different viruses can cause cold symptoms, but the great majority are not associated with anosmia, hyposmia, and CNS involvement. Besides, most previous reviews, similar to what is observed in the context of the COVID-19 pandemic, have found that POVD is more common in women. Still, olfactory dysfunction could occur only after infection by a specific virus with peculiar neurotrophic proprieties, and when the host had some predisposing factors, virus may invade the CNS (16, 17). However, the pathogenesis of sensory loss and associated predisposing factors after viral infections are not well-characterized, and unfortunately, most patients were investigated when the virus was no longer detectable (17). The SARS-CoV-2 pandemic has turned our attention on POVD again.

Now, we know the straight association between anosmia and SARS-CoV-2. Thus, we should take advantage to better understand the pathogenesis of POVD and the neuroinvasive potential of the virus through the olfactory neuroepithelium (ONE) and olfactory pathway.

After the intranasal inoculation of several viruses, previous animal studies have shown central olfactory damage and damage to deeper areas of CNS (18, 19).

Therefore, the question is whether the olfactory dysfunction in COVID-19 and other viral infection arise from peripheral OR damage as a result of local inflammation or involvement of central olfactory pathways, or a combination of both (17).

Previous studies on POVD highlight direct evidence of a broad spectrum of epithelial damage, from the reduced number of ORs to abnormal dendrites that did not reach the epithelial surface or that were lacking sensory cilia to decreased nerve bundles or substitution of ONE with metaplastic squamous epithelium (20–23).

In contrast with these studies, Chung et al., analyzing the nasal mucosae of patients affected by SARS-CoV-2, showed minimal inflammatory changes represented by mild infiltrations of lymphocytes, plasma cells, and occasional neutrophils localized in the stroma but without detailed characterization of neuroepithelium alterations (12).

In a recent study, local TNF-α and IL-1β levels were assessed in COVID-19 patients. TNF-α was significantly increased in the olfactory epithelium of the COVID-19 group compared to the control group. However, no differences in IL-1β were seen between groups. In the authors' opinion, this evidence implies that inflammation can lead to OR impairment, and according to a previous study, this impairment arises from inflammation, which can damage olfactory neurons (24).

On the contrary, Kim and Hong demonstrated that persistent PVOD is associated with decreased metabolism in specific brain regions where the olfactory stimuli are processed and integrated, suggesting that anosmia is, in some cases, caused by a central injury mechanism (25). Virally induced damage of OB and other brain areas was highlighted through magnetic resonance imaging correlating olfactory function with OB volume (26).

Retrograde transport from the nasal mucosa to the brain has been recently hypothesized for SARS-CoV-2 (27–30) and previously described for SARS-CoV1 and HCoV-OC43 found in specific brain areas of infected patients (31–33) probably climbing *via* the olfactory nerves, as already shown in mice (34). Furthermore, once in the brain, HCoV-OC43 can disseminate from the OB to other regions of the brain, including the cortex and the hippocampus, from which it appears to spread by a trans-neuronal route before it eventually reaches the brainstem and spinal cord. These results suggest that coronaviruses may also invade the human CNS from the external environment through the neuroepithelium of the olfactory nerve and OB, before infecting the resident cells of the brain, and potentially the spinal cord. Mori et al. also reviewed these same observations for some neuroinvasive human viruses such as influenza virus and Herpes simplex virus (HSV) (35). Presumably, SARS-CoV-2 can involve the olfaction through a central mechanism also.

However, a recent study highlighted that olfactory epithelial cells, but not OR neurons (e.g., horizontal basal cells and supporting cells), express ACE2, the primary SARS-CoV-2 receptors (36). Therefore, virus could use alternative receptors to directly enter into OR neurons or an alternative pathway for OB involvement. The OB may be the first site of CNS involvement by neurotropic viruses.

## Immunological Role of the OB

Olfaction, although not indispensable to the survival, is a crucial sense that induces several feedback processes, also unconscious in response to the molecular sampling of the environment. These processes are very complex, as well as the anatomical substrates that allow them. From odor receptors, the stimuli converge in the OB and then, through a multitude of projections, reach the higher brain regions, including the amygdala, septal nuclei, pre-pyriform cortex, the entorhinal cortex, hippocampus and the subiculum, thalamus, and the frontal cortex. These bidirectional connections provide a unique dynamic system (37). Considering the intricate interactions between the immune system and the CNS and the complexity of the relationship between CNS and the olfactory system, it does not surprise the relevance of smell for immunological investigation. Since OB, regularly exposed to the external environment, is considered an immune organ that prevents the invasion of viruses into the CNS (3), its dysfunction may be a concomitant factor that predisposes to a worse outcome in respiratory virus infections when his immunological function is impaired or disrupted as a result of aging or some pathological processes. In many animal studies concerning depression, olfactory bulbectomy is commonly used. Unexpectedly, a variety of immune abnormalities may be observed in the olfactory bulbectomized mice: reduced neutrophil phagocytosis, lymphocyte mitogenesis, lymphocyte number, and negative acute-phase proteins and increased leukocyte adhesiveness/aggregation, monocyte phagocytosis, neutrophil number, and positive acute-phase proteins. In addition, after bulbectomy, increases in serum IL-1β concentration and PGE2, while basal anti-inflammatory cytokine IL-10 concentration is suppressed, may be observed (38, 39).

These observations suggest that the olfactory system is intricately related to immunological function, and perturbations in the immunological system and in the olfactory system may be significant to each other (37).

Viral infection of the CNS is a rare condition and efficient mechanisms must be in place in the OB to protect the CNS from viral spread: Kalinke et al. showed that after intranasal instillation of VSV in mice with selective type I interferon receptor depletion only in neuroectodermal cells, the virus moved *via* the olfactory nerves to the OB and further spread over the whole CNS. On the contrary, control mice infected with the same virus showed infection of olfactory nerves, but within the OB, the virus was arrested in the glomerular layer (40).

This experiment highlighted in the OB a type I IFN-dependent mechanism that efficiently inhibited virus spread. After exposure to viruses, expression of MHC I and II, pattern recognition receptors (PRRs), and type 1 interferon (IFN-I) is increased in astrocytes, microglia, and ONE. The increase in INF-I and the rapid infiltration of both CD4+ and CD8+ T cells decrease viral load in the OB (3). Therefore, inflammatory cell infiltration in OB, while possibly involved in the development of anosmia, contributes to the viral spread arrest. Unfortunately, it is unknown whether type I IFN stimulation of olfactory neurons affects axonal virus spread and infection of the CNS.

In an interesting recent work, according to a previous study that demonstrated selective expression of interferon-gamma in sustentacular cells inducing anosmia without damage to the neuroepithelia, the authors hypothesized that IFNs, or other cytokines, can activate an antiviral response within the OR neurons that suppress OR expression. They also demonstrated that interferon signaling correlates with OR neuron dysfunction (41). If this IFN-I antiviral response model is confirmed in patients with COVID-19 infection, ORs may have an essential role in the virus mechanisms of cell infection.

The OR neuron cell body is located in the olfactory epithelium, whereas their axons project into the OB, and the virus can readily spread within the OB in an anterograde manner. To further move to other brain areas, the virus can spread trans-synaptically using retrograde and anterograde transport. Considering the capability of some coronaviruses to spread from the lower respiratory tract by a synapse connection to the medullary cardiorespiratory center (partially responsible for the acute respiratory failure of COVID-19 patients) (34), it is possible to hypothesize an inverse path: SARS-CoV-2 could spread from OB to the CNS to periphery through nerve endings of the lower respiratory tract. Obviously, the virus can enter the CNS also *via* non-olfactory paths. It was shown that, after intranasal VSV instillation, the olfactory route is preferentially used for CNS entry. Only if OR neurons are destroyed, are alternative entry paths, such as *via* the cerebrospinal fluid or the trigeminal nerve or blood, used (27). The local microenvironment of distinct brain regions may be critical to determine virus permissiveness. The neurological involvement can occur independently of the respiratory system and coronaviruses could infect brainstem neurons responsible for the cardiorespiratory regulation, resulting in hypoxia (42). It would be interesting to know if OR neurons are infected, microscopic alteration of OB, and grading of corresponding olfaction alteration, under conditions of subclinical infections vs. severe disease. Unfortunately, to date, only a few studies documented OB involvement. Politi et al. show a first report of *in vivo* human brain involved in a patient with COVID-19 showing an MRI signal alteration compatible with viral brain invasion in the OB and cortical region (i.e., posterior gyrus rectus) associated with anosmia 3 days later his onset. No brain abnormalities were seen in other patients with COVID-19 presenting anosmia who underwent brain MRI in this and other studies (43). Nevertheless, COVID-19 patients in need of admission in an intensive care unit, not investigated adequately for anosmia, could have direct involvement of higher CNS center detectable by MRI. Li et al. reported a 21-years-old male with a 5-days loss of smell, initially without other symptoms and respiratory tract involvement. On the day of discharge, after 23 days of hospitalization with partial recovery of the sense of smell, brain magnetic resonance imaging (MRI) showed smaller right olfactory blub and linear hyperintensities inside bilateral olfactory nerves, suggestive of bilateral olfactory neuropathy (44). A recent case series highlighted the abnormal intensity of the OBs in five COVID-19 adult patients, three of whom had anosmia, maybe due to abnormal enhancement or microbleeding because they only underwent the sequence after injection of gadolinium in fat-suppressed T1WI (45).

## SARS-CoV-2–Host Interaction and ORs

Virus entry into specific cells and virus spread in different organs depend on virus–receptor interaction and the involvement of coreceptors. Using multiple receptors might be advantageous for virus spread to various organs. Some viruses can use more than one receptor or mutate their envelope proteins by acquiring the ability to bind different receptors or coreceptors. Thus, in the beginning, infection usually has a minor impact on the host, while the subsequent replication of the virus may significantly damage secondary organs (46, 47). Admittedly, SARS-CoV-2 causes a broad spectrum of clinical manifestations characterized in most cases in significant pulmonary damage. Still, other organs may be involved, including the heart, kidney, liver, gastrointestinal tract, gonadal function in males, and, as recently emphasized, the CNS (7).

The angiotensin-converting enzyme 2 (ACE2) is considered the primary receptor for cellular entry for SARS-CoV-2 (29). This knowledge has driven researchers to investigate the expression of ACE2 in different tissues to relate it to the clinical phenotypes of the disease. More specifically, regarding the neurological involvement, glial cells and neurons have been reported to express ACE2 receptors, and previous studies have shown that SARS-CoV may cause neuronal death in mice by invading the brain *via* the olfactory epithelium (48). Autopsy findings in humans have also demonstrated the presence of SARS-CoV by electron microscopy, immunohistochemistry, and real-time reverse transcription-PCR into the CNS (48). The confirmed entry mechanism of coronaviruses (SARS-CoV, MERS-CoV, and hCoVs) is mediated by the Spike protein of the virus that directly binds cell receptors (ACE2) and, after being cleaved by a protease (TMPRSS2), allows membrane fusion (49–52). Thus, the coronavirus entry into cells seems to be conditioned not only by the expression of the receptor but also by the protease expression. Therefore, several studies have performed *ACE2* and *TMPRSS2* co-expression profiles into healthy human tissues to identify possible target organs. However, a recent literature review has underlined several limitations of most studies, highlighting that these two proteins alone cannot explain all the clinical observations. Indeed, it was noted that several cell lines, which do not express *ACE2* RNA, can be infected by SARS-CoV-2 and that *ACE2* expression could not be detected in healthy individual organs, including lung, bronchus, nasopharynx, esophagus, liver, and stomach, contrasting to clinical data of SARS-CoV-2 infection. It was also observed, for instance, that cardiomyocytes express ACE2 but do not appear to express *TMPRSS2/4*, and some studies reported *ACE2* expression in various brain cells, but *ACE2* and *TMPRSS2/4* were rarely co-expressed within the same cells. Besides, a recent study failed to detect ACE2 expression in mature OR neurons at either the transcript or protein levels and in neurons in the OB. A detailed survey of nasal epithelium did not detect TMPRSS2 in the neuronal component (53, 54). Together, these observations suggest that our understanding of SARS-CoV-2 cellular tropism is still insufficient, and maybe SARS-CoV-2 could bind alternative receptors to enter into several cells. We have hypothesized that these alternative receptors need to have specific properties, including being highly conserved between species, presenting polymorphism, ubiquitous expression in human organs, and altered expression depending on age, sex, and comorbidity.

Our attention was focused on a family of receptors, “sensory G-protein coupled receptors (GPCRs)” (55), still poorly understood, suffering from the bias due to their discovery as specialized receptors expressed on sensory neurons only in the nose: ORs.

ORs represent the largest gene family in the human genome (418 genes classified into 18 families). ORs are divided into two classes, class I receptors and class II receptors, based on the species they were initially identified: aquatic and terrestrial animals, respectively. In humans, all class I genes are located on chromosome 1, while class II genes are located on all chromosomes except chromosomes 20 and Y (40, 56).

ORs are expressed throughout the body, and their expression in non-olfactory tissues has been documented for more than 20 years, but the most significant part is still “orphans” of ligands. Their functional roles were unknown, but many studies have demonstrated that these G-protein-coupled receptors are involved in various cellular processes (40, 57).

### Highly Conserved Receptors

Olfaction is one of the most developed senses in animals [including bats, the natural reservoir of SARS-CoV-2 (58)], and evolutionary conservation was also demonstrated for ectopic OR between mouse, rat, and humans (40, 59–61).

OR proteins are composed of highly conserved (each family having >40% sequence identity) amino acid motifs that distinguish them from other GPCRs and some highly conserved motifs with other non-OR GPCRs. These OR residue sequences seem to have specific functional activities. In analogy to other Class A GPCRs, each OR has seven transmembrane domains (TM1–TM7) connected by extracellular and intracellular loops. Additionally, there is an extracellular N-terminal chain and an intracellular C-terminal chain that, together with TM4, TM5, and the central region of TM3, are highly variable, participating in ligand binding. The fact that some amino acid sequences have been evolutionarily conserved across species implies that they may have critical roles (61).

### Polymorphism

There is a wide variability of functional OR genes among different people. Recent studies, genotyping 51 odor receptor loci in 189 individuals of several ethnic origins, found 178 functionally different genomes. Additional variation in the population may come from differences in gene expression. Indeed, other experiments have found that the expressed OR repertoire of any pair of individuals differs by at least 14%, suggesting that polymorphisms also exist in the promoter and other regulatory regions. Furthermore, variation in the copy number of OR genes contributes significantly to individual olfactory abilities (55, 61, 62).

### Ubiquitous Expression in Human Organs

ORs are detected in migrating neural crest, smooth muscle, endothelial precursors and vascular endothelium, endocardial cells, neuroepithelium, and ocular tissues. ORs were found in various additional non-olfactory tissues, including the prostate, tongue, erythroid cells, heart, skeletal muscle, skin, lung, testis, placenta, embryo, kidney, liver, brain, and gut (40). Moreover, ORs are detected in cancerous tissues of the liver, prostate, and intestine (63, 64). In non-olfactory tissues, several ORs are co-expressed in the same cell, as demonstrated for the B- and T-lymphocytes, polymorphonuclear leukocytes, and human sperm cells, while in the olfactory system, only one allele of OR gene is expressed in each olfactory neuron (40).

Physiological functions of non-olfactory ORs are not entirely understood and seem to be unrelated to the olfactory system in diverse cell types. For instance, renal and cardiovascular ORs regulate blood pressure, ORs on airway smooth muscle decrease remodeling and proliferation, while exposure of the airways to γ/LPS resulted in markedly increased OR expression (65). ORs on pulmonary macrophages, induced by IFN-γ and LPS, may contribute to innate immune response (66).

### Altered Expression Depends on Age, Sex, and Comorbidity

A large number of OR genes appear to be detectable only after birth. In mice, experiments demonstrated that the expression level of ORs could be classified into different patterns that reach a peak at different ages. For example, some ORs reach a height of expression between the 10 and 20th day of life and then reduce to a low level while other ORs reach a peak at the 10th day of life and continue to be expressed at a high level until 18 months. In the authors' opinion, these expression patterns may correlate with their functions in each life stage, such as nursing or reproductive cycle (61). Additionally, there is a high incidence of age-related olfactory dysfunction supported by histological evidence in the olfactory epithelium (67).

Interestingly, sex differences in olfaction are highlighted in a meta-analysis: the female OB presents more dense microcircuits and slower aging than males (68). Another study reveals that mRNA levels of sex steroid, GnRH receptor, and aromatase in the OB vary with sex, social status in males, and females' reproductive condition. In the authors' opinion, these observation highlights that during the reproductive cycle, OR expression level may change to fine-tune the olfactory system, suggesting the hypothesis that the changes in receptor levels could be an essential mechanism for regulating reproductive, social, and seasonal plasticity in olfactory perception (69). In another work, a sex difference in the absolute number of total, neuronal, and non-neuronal cells was demonstrated, favoring women by 40–50%, also after correction for mass. Thus, it was hypothesized that quantitative cellular differences may have functional impact (70). ORs in non-olfactory tissue are correlated with the development of several diseases such as glucose homeostasis in diabetes, tumor cell proliferation, apoptosis, metastasis, and invasiveness. Some ORs seem to accelerate obesity development, angiogenesis, and tissue regeneration and to initiate hypoxic ventilatory responses (71).

Concerning the CNS, in the adult human brain, several ORs are expressed in neurons of the neocortex, hippocampus, dentate gyrus, striatum, thalamus, nuclei of the basal forebrain, hypothalamus, nuclei of the brainstem, cerebellar cortex, dentate nucleus, and neurons of the spinal cord. ORs have also been reported in the autonomic nervous system and murine sensory ganglia. Their functions and kinetic expressions are still unknown (65). Interestingly, OR gene expression into the CNS is altered in several neurodegenerative diseases, including Parkinson's disease (PD) and Alzheimer's disease (AD). For example, it was demonstrated that the mRNA encoding for some ORs increases with age in the cortex and hippocampus of wild-type and transgenic Alzheimer's disease-like mice; nonetheless, transcript expression of the same ORs is impaired in the brain of Alzheimer's disease-like mice. Besides, in transgenic Alzheimer's disease-like mice, ORs are observed near amyloid plaques (65, 72).

All these features make ORs ideal viral receptors and could also contribute to explain the broad spectrum and wide interindividual variability of clinical manifestations in COVID-19.

We have no data to support our hypothesis. Our knowledge of the ORs is insufficient in terms of physiological and pathological functions, intra-individual diversity during life, epigenetic processes acting on ORs expression, and above all ORs ligands within the different cell types. Identifying these cell-surface receptors as required for viral infection, given their peculiar characteristics, may be necessary for developing antiviral therapies and effective vaccines. To our knowledge, the only literature data supporting the possible involvement of ORs in virus entry into a cell is on OR14I1 as a receptor for HCMV infection. This OR is required for HCMV attachment, entry, and infection of epithelial cells, revealing previously neglected targets for vaccines and antiviral therapies (73, 74). Unfortunately, like many others, OR14I1 is an “orphan receptor” without known ligand. As noted concerning HCMV, these findings do not exclude roles for other receptors and coreceptors during infection but answer questions regarding epithelial tropism of HCMV and offer alternative opportunity to develop antiviral strategies for the management and transmission of the disease. The same could happen in COVID-19 and other infectious diseases if only ORs were considered.

## Hypothesis Statements and Data Discussion

Understanding the mechanisms behind COVID-19-related olfactory dysfunction will require further investigation to delineate his prognostic value concerning coronavirus neuroinvasion, immune reaction, and virus spread from the nasal cavity to other distant organs. However, considering all the data above described, it is possible to propose several hypotheses.

Since anosmia has been observed generally in the absence of cold and rhinosinusitis and considering the reported persistent hyposmia also detected after clinical recovery, we may hypothesize the prevalence of sensorineural dysfunction.

Defining the role of local inflammatory mediators in host defense and tissue damage of ONE may explain the mechanism of COVID-19-related anosmia. Indeed, in non-infected cells, the interaction between virus and receptor may induce defense mechanisms resulting in cytokine secretion (i.e., interferons), apoptosis, and innate immune response, which can have a significant impact on the development of disease both locally and at the systemic level.

Adults with severe disease have a depletion of the B-cell compartment (75), and levels of serum IL-2R, IL-6, IL-10, and TNF-α are higher than in moderate cases. The absolute number of CD4+ T and CD8+ T cells decreased in nearly all the patients and were markedly lower in severe cases than moderate ones. The expression of IFN-γ by CD4+ T cells tended to be lower in severe cases (14.1%) than in moderate ones (22.8%) (76).

On the contrary, in the pediatric population, recent studies showed that an early polyclonal B-cell response (75) augmented percentage of CD3+, CD4+, and CD8+ lymphocytes related to increased levels of IL-6, IL-10, and IFN-γ (76). These data observed at the systemic level may reflect what locally happens when, in the elderly, IFN-dependent OB defense mechanism is not efficient, causing OB barrier to overcome, subsequently, virus spread and worse outcome.

It may be suggested, considering the above-discussed interaction between olfactory and immunological systems, that the nasal epithelium and OB may be one of the first battlefields between SARS-CoV-2 and host; the outcome of this battle may be critical for the pathological development of COVID-19. Considering the OB as an immune organ, if the local fight against SARS-CoV-2 is successful, the damage remains localized, leading to anosmia, as in the case of women and younger patients; conversely, the virus can spread and replicate in the upper olfactory sites causing central anosmia or directly invading the CNS. The prevalence of chronic olfactory impairment increases with age. Olfactory deficit affects up to 50% of people ages ≥65 years and >80% of people ages ≥80 (77). It is clear that COVID-19 causes more severe complications in patients with advanced chronological age. The age-related dysregulation of ONE and OB homeostasis might contribute to more severe manifestations of COVID-19 in the elderly, likely due to immunosenescence. The spread of the virus and the neuroinvasive potential have been proposed according to the known routes of SARS-CoV and a growing body of findings specific for SARS-CoV-2. Since the basal expression level of receptors determines, at least in part, the tropism of the virus, identifying the kind of receptor involved is crucial to predict which tissues are probably involved in infection and to guide research toward new prevention and therapeutic strategies. In this context, considering the contrasting data concerning ACE2 expression in human tissue that cannot entirely explain the wide spectrum of clinical manifestation, we hypothesize that SARS-CoV-2 could use ORs as ideal alternative entrance receptors as already demonstrated for HCMV.

This hypothesis may have the power to attract the attention of the broader community of scientists and neuroscientists on the olfactory system to investigate the biological significance of these neglected receptors in sickness and health.

Besides the acute neurological involvement of SARS CoV-2 infection, there are many overlaps between SARS-CoV-2-related manifestations and OR-related disease. For instance, it has been shown in animal and human studies that coronaviruses could be implicated in the pathogenesis of Parkinson's disease, acute disseminated encephalomyelitis, multiple sclerosis, and other neurodegenerative diseases, as well as for ORs. Further monitoring for long-term sequelae may reveal viral contribution in pathophysiology or increased risk for neuroinflammatory and neurodegenerative diseases and the possible link with OR dysregulation or damage.

In light of these observations, the role of ORs and OB in COVID-19 infection could be significant, explaining at least in part the age- and sex-related differences in the clinical course.

In conclusion, from anosmia onset in SARS-CoV-2-positive patients, a precise timing for the olfactory route climbing by the virus can be speculated. In this window period, a potential early intervention could change the disease's course, supporting natural defenses when they lack, as a result of age, sex, or other genetic backgrounds. Since PAMPs (pathogen-associated molecular patterns) can improve the up-regulation of IFN, it could be hypnotized to use immune-stimulatory molecules to increase the ability to fight the infection. Simultaneously, the use of other topical pharmacological agents (i.e., antiviral drugs and “molecular competitor binding ORs”) could be helpful.

## Conclusion

The evidence of OB involvement in COVID-19 remains scarce, but the knowledge of this different way of spreading could lead to significant developments in the management of SARS-CoV-2.

Magnetic resonance imaging cannot be an early detector tool in all COVID-19 patients with anosmia. However, the latest evidence on the CNS involvement, beyond the anosmia, could justify it as a valid indication in high-risk patients. Autopsies of the COVID-19 patients, detailed neurological investigation, and attempts to isolate SARS-CoV-2 from OB and neuronal tissue can clarify the role of this novel coronavirus in the mortality linked to neurological involvement. Existing studies to assess the incidence of anosmia and related immunological patterns are limited; therefore, investigating the local cytokine composition at the onset of symptoms could be useful.

In conclusion, we invite to focus on anosmia in each patient suspected of infection or with a positive swab for SARS-CoV-2. Future studies should evaluate the degree of functional impairment (grading), central/peripheral anosmia (localization), and the temporal course (evolution) through MRI and olfactory tests, perhaps through standardized workup protocol to explore this issue better.

## Data Availability Statement

The original contributions presented in the study are included in the article/supplementary material, further inquiries can be directed to the corresponding author/s.

## Author Contributions

All persons who meet authorship ICMJE criteria are listed as authors, and all authors certify that they have participated equally in the work to take public responsibility for the content, including participation in the concept, design, analysis, writing, or revision of the manuscript. Furthermore, each author certifies that this material or similar material has not been and will not be submitted to or published in any other publication.

## Conflict of Interest

The authors declare that the research was conducted in the absence of any commercial or financial relationships that could be construed as a potential conflict of interest.
